# Knowledge and Attitudes of Turkish Physicians towards Human Monkeypox Disease and Related Vaccination: A Cross-Sectional Study

**DOI:** 10.3390/vaccines11010019

**Published:** 2022-12-21

**Authors:** Taha Koray Sahin, Enes Erul, Melek Seren Aksun, Meliha Cagla Sonmezer, Serhat Unal, Murat Akova

**Affiliations:** 1 Internal Medicine Department, Faculty of Medicine, Hacettepe University, Ankara 06100, Turkey; 2 Infectious Diseases and Clinical Microbiology Department, Faculty of Medicine, Hacettepe University, Ankara 06100, Turkey

**Keywords:** human monkeypox, disease, knowledge, health care workers, vaccination, Turkey

## Abstract

Background: In May 2022, the monkeypox virus outbreak in multiple countries on various continents marked a potential resurgence of the disease as a global health issue. Considering the crucial role of physicians in mitigating the monkeypox outbreak, we sought to evaluate physicians’ knowledge, attitude, concerns, and vaccine acceptance for monkeypox, in the shadow of the COVID-19 pandemic. Methods: A large-scale, cross-sectional survey was conducted among 283 physicians between 20 August–2 September 2022, in Turkey. The participants’ sociodemographic characteristics, knowledge, attitudes, concerns, and vaccine acceptance toward monkeypox infection were collected via a questionnaire. Results: Our study revealed that 32.5% of physicians achieved a good level of knowledge; similarly, 31.4% of the physicians planned to have the monkeypox vaccine. Multivariate binary logistic regression analysis showed that female physicians (*p* = 0.031) and older people (≥30 vs. <30) were more likely to be knowledgeable about monkeypox (*p* = 0.007). We found that participants from divisions of internal medicine (*p* = 0.033) who knew about the monkeypox disease during medical school or residency (*p* = 0.005) and were previously exposed to COVID-19 disease (*p* = 0.005) were more likely to have a good knowledge score of monkeypox. We also found that physicians with a good knowledge score were more worried about monkeypox compared to COVID-19 (AOR: 2.22; 95% CI:1.13–4.33; *p* = 0.019). Additionally, those who had information on monkeypox during medical education (AOR = 2.16, 95% CI = 1.10–4.21; *p* = 0.024) were more likely to receive the smallpox vaccine to prevent monkeypox viral infection when available. Conclusions: The present study pointed out that physicians in Turkey have unsatisfactory levels of knowledge about the emerging monkeypox. This study results can impede attempts to detect and manage cases of monkeypox and should be addressed through appropriate and timely awareness and educational programs, alerts, and seminars. These might serve as the basis for policymakers’ decisions about promoting national monkeypox vaccination strategies and addressing potential vaccine hesitancy and misinformation when needed.

## 1. Introduction

Human monkeypox is a rare zoonotic infectious disease caused by the monkeypox DNA virus belonging to the orthopoxvirus of the poxviridae family, including smallpox. Monkeypox is spread primarily through respiratory droplets, body fluids, and close contact with skin lesions of the infected animals [1]. The disease can also be transmitted to humans through close physical, face-to-face, or skin-to-skin contact [2]. Regarding the clinical manifestations, human monkeypox and smallpox share very similar signs and symptoms, albeit the former causes less severe disease and has a better outcome [3]. The symptoms and signs include fever, skin rash, generalized headache, myalgia, and back pain. The main difference between monkeypox and smallpox is that the latter does not cause lymphadenopathy [4].

The first human monkeypox case was identified in 1970 when a 9-month-old child developed an illness similar to smallpox in the Democratic Republic of Congo, where smallpox had been eliminated in 1968 [5]. Due to the limited secondary spread of these travel-associated cases and outbreaks outside of Africa, human-to-human transmission has been deemed inefficient [6,7]. At the beginning of the 2000s, monkeypox infections popped up and spread from endemic to non-endemic countries. In late spring 2003, the first outbreak of monkeypox outside the African continent was reported in the midwestern United States and was associated with exposure to infected prairie dogs [8]. In late spring of 2022, an unprecedented but not unexpected outbreak of human monkeypox began to spread in Europe and the USA among individuals who had not traveled to endemic areas. Autochthonous cases of monkeypox infection were initially confirmed in the United Kingdom in early May 2022 and subsequently throughout Europe [9,10]. Turkey reported the first confirmed case of the monkeypox virus in a 37-year-old immunocompromised patient on 30 June 2022 [11]. The World Health Organization (WHO) declared monkeypox as a “Public Health Emergency of International Concern” on 23 July 2022, as a result of more than 5000 cases officially notified in more than 50 countries across 5 regions as of the beginning of May 2022 [12]. As of September 9, it is covering 107 different nations and infecting 65,415 people; 64,835 cases were reported across 99 countries in America, Asia, Europe, and Australia, many of which have not previously reported monkeypox cases, and 580 cases were reported from eight endemic African countries [13].

However, the prevention and treatment of monkeypox remain challenging in endemic areas. Isolation and vaccines can be used as preventive measures to prevent the spread of the disease from human to human. Although vaccination against smallpox had been shown to provide 85% protection against the monkeypox virus [14], smallpox vaccination has ceased since 1980 [15], after the declaration of disease by the WHO. In addition, tecovirimat, an antiviral medication for smallpox, was approved by the European Medical Association for use in treating monkeypox, based on both animal and human research [16]. According to a WHO report, one of the obstacles in preventing the resurgence of monkeypox was a lack of knowledge about monkeypox, especially among healthcare workers (HCWs) [17]. Another recent study demonstrated that only 18.6% of HCWs reported having some knowledge of managing monkeypox [18]. Since Turkey is a popular tourist destination, physicians must be prepared and knowledgeable about cases of monkeypox to develop a global strategy to combat the emergence of monkeypox. However, there is no documented evidence regarding the knowledge and attitudes of Turkish physicians toward human monkeypox and related vaccination. Hence, we sought to evaluate Turkish physicians’ knowledge, attitude, and concerns about human monkeypox at Hacettepe University in Ankara, Turkey. Moreover, we assessed whether physicians would like to receive the smallpox vaccine to prevent monkeypox viral infection and the potential factors that influence this.

## 2. Materials and Methods

### 2.1. Setting, Data Collection, and Study Design

We conducted a descriptive, cross-sectional study between 20 August–2 September 2022, among physicians currently working at the Hacettepe University Hospital, Ankara, Turkey. Our institution is an 850-bed capacity tertiary care university hospital.

Google Forms was used as a platform to construct automatically hosted online questionnaires via a specific URL. The participants were included with an invitation letter containing a summary of the study, distributed via links on social media platforms, including WhatsApp, Instagram, and Facebook. The web-based survey was preferred to provide prompt, thorough, and high-yield data collection and interpretation. Data were obtained from completed questionnaires because a response to every item was mandatory to handle the problem of nonresponse bias. We included all the physicians currently working at the Hacettepe University Hospital in the study. Exclusion criteria were: (1) undergraduate medical students from 1st–5th year of medical school; (2) internet non-users and; (3) physicians who refused to provide informed consent.

The Raosoft calculator was used to determine the minimum required sample size. As no previous studies in Turkey have investigated physicians’ knowledge and attitudes about monkeypox, a conservative estimate of 50 percent was used. A calculated sample size of at least 278 complete respondents would provide a level of confidence of 95% with an error margin of 5% among physicians currently working at the Hacettepe University Hospital.

### 2.2. Ethical Approval

The study was approved by the Research Ethics Committee; Faculty of Medicine; Hacettepe University (GO 22/808). Participants were informed that their responses would be used for research by participating in the survey. Participants were prompted to confirm that they consented to participate and met the eligibility criteria at the beginning of the study. Additionally, participants were assured of the confidentiality and anonymity of their survey responses

### 2.3. Overview of the Questionnaire and Study Variables

The survey was adapted and developed based on previously published studies on COVID-19 and the new monkeypox outbreak [17,19,20,21]. The questionnaire was composed of five sections: socio-demographic data, knowledge test, attitudes, vaccine acceptance/hesitancy, and anxiety about monkeypox. Since all physicians were native Turkish speakers, all questions were written in Turkish. The English-translated version of the questionnaire is available in Supplementary Table S1. The pretest was conducted among 10 participants who were not included in the study before launching a survey. Based on their feedback, we made improvements to the questions and structure of the survey that might lead to biased responses.

The survey begins with general information and socio-demographic data from the participants. It includes age, gender, medical specialty, medical practice experience, knowledge of monkeypox during medical education, previous COVID-19 infection, degrees of worries about monkeypox compared to COVID-19, whether COVID-19 vaccination shots were received in the past, and monkeypox-related information sources. The second section covered monkeypox knowledge, which included 20 statements on monkeypox (Table 1). The possible responses to each knowledge item were (correct vs. incorrect vs. I do not know). A monkeypox knowledge score was then calculated by adding 1 to the total score for each correct answer.

In contrast, an incorrect answer or a “don’t know” response added 0 to the total score (potential range 0 to 20). Poor and good levels of knowledge for the overall assessment were defined at a level of 70% correct responses [17]. In the next section, in order to assess the attitude level towards monkeypox, we set a 5-item section, with three-point Likert scale possible responses as follows: (1) disagree (0), (2) undecided (1), (3) agree (2) (Supplementary Table S2). A positive attitude was defined as having a score more than or equal to the average value of the participants’ scores, and a negative attitude as having a score below the average. The fourth section covered queries related to the participants’ willingness to be vaccinated against monkeypox. Participants were asked about the likelihood of getting the smallpox vaccine to prevent monkeypox viral infection once made available. Responses were categorized as “willing” if the respondent selected agree and “unwilling” if they chose undecided or disagreed. Variables surveyed included the concerns regarding monkeypox vaccination safety and the efficacy of smallpox and chickenpox vaccines against monkeypox disease. Finally, the generalized anxiety disorder (GAD-7) score, an easily applicable and validated 7-item tool, was used to measure general anxiety symptoms [22]. The GAD-7 score assesses how often participants have encountered each anxiety symptom over the last two weeks. It consists of seven items: an inability to manage worrying, restlessness, experiencing nervous symptoms, annoyance, worrying about many things, trouble relaxing, and fear that something terrible might happen. Items are scored using a four-point scale indicating symptom frequency, ranging from 0 = “not at all” to 3 = “nearly every day”. GAD-7 scores were totaled and categorized into four severity groups as follows: 0–4 minimal anxiety, 5–9 mild anxiety, 10–14 moderate anxiety, and 15–21 severe anxiety. Using the cut-off score of 10, GAD-7 identified anxiety cases with 82% specificity and 89% sensitivity [22].

### 2.4. Statistical Analysis

All the data analyses were carried out using IBM SPSS Statistics v. 24 (IBM Corporation, Armonk, NY, USA). For categorical variables, baseline characteristics of participants were given as frequencies with percentages, while continuous variables were presented as average (standard deviation) and median (interquartile range; IQR). The Kolmogorov–Smirnov test and the histogram were applied to test the normality of distribution, and the Levene’s test was used to test the homogeneity of statistical variance assumption. In addition, a chi-square test was performed to find the potential relationships among variables and participants’ knowledge score, attitudes score, and willingness to obtain the smallpox vaccination to prevent monkeypox virus infection. After, bivariate logistic regression analysis was performed, and variables with *p* < 0.25 and other variables of known clinical relevance were retained for multivariable logistic regression analysis. From the multivariable analysis, variables with a significance level of *p* < 0.05 with adjusted odds ratio (AOR) at 95% CI were taken as statistically significant factors that were independently associated with a good knowledge score and willingness to get the monkeypox vaccine.

## 3. Results

### 3.1. Baseline Characteristics and Demographic Data

A total of 283 physicians were enrolled in the study. Participants’ sociodemographic characteristics are shown in Table 2. Most participants were between the ages of 18–29 (52.7%). More than half of them were female (58.7%) and physicians in the Division of Internal Medical Sciences, including infectious disease, internal medicine, and pediatrics (60.8%). Approximately 38.9% of the study participants had 1–5 years of medical experience. Nearly half of the participants learned about monkeypox virus-related information from social media (51.9%), followed by research articles (48.8%). About one fifth of participants reported getting information on monkeypox during medical education (21.2%). Although 71.7% had received four or more COVID-19 vaccines, 66.4% of participants reported being previously infected with COVID-19.

### 3.2. Knowledge and Attitude of Study Participants about Monkeypox Disease

**Figure 1:**
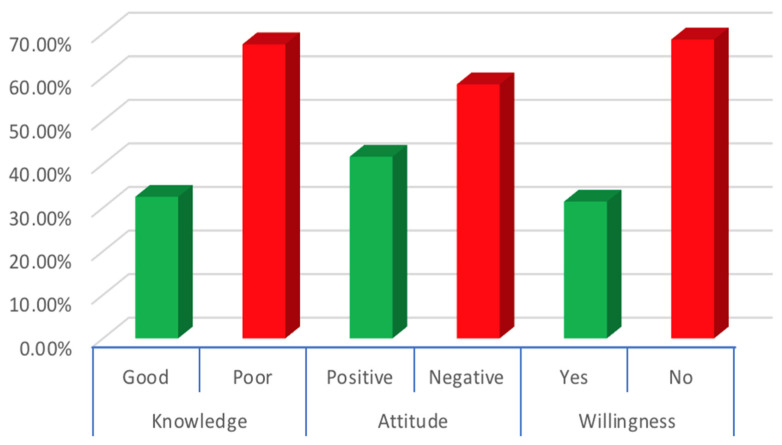
Percentage distributions of knowledge (good and poor), attitude (good and poor), and vaccine willingness (yes and no) for monkeypox among Turkish physicians.

The participant’s knowledge level was evaluated based on 20 questions assessing participants’ knowledge, and the average knowledge score was calculated at 11.26 ± 3.67 (mean ± SD) out of 20. Similarly, the participants’ attitudes were graded based on 5 questions, and the average attitude score of the participants was measured at 6.10 ± 2.05 (Table 3). Accordingly, 92 participants (32.5%) achieved a good level of knowledge based on a 70% cut-off (i.e., 14 questions correct), whereas 118 participants (41.7%) exhibited a positive attitude regarding monkeypox (Figure 1).

Table 1 displays participants’ responses to questions assessing their knowledge of monkeypox. The overall level of knowledge about human monkeypox was poor, with just five questions receiving correct responses at a rate greater than 70%. Most participants were aware that this was not a bacterial infection. It does not only affect men and is not a new infection that emerged in 2022. Even if more than half correctly responded that monkeypox was not sexually transmissible (52.7%), the minority correctly knew that monkeypox might spread to people by scratches and bites from infected animals (42%). When assessed about monkeypox symptoms knowledge, the vast majority (86.9%) correctly agreed that papules and vesicles were a symptom, but less (41%) accurately knew that lymphadenopathy was distinctive for monkeypox and smallpox.

Regarding vaccination status, a minority of participants reportedly knew that a smallpox vaccine for vaccination against monkeypox was available (39.6%). However, even less (20.5%) acknowledged that people could be vaccinated after exposure to monkeypox. Notably, more than half of the participants were aware of the lack of specific vaccines and treatments for monkeypox (58.3% and 60.8%, respectively).

The results of chi-square analysis revealed that several factors, including gender (*p* < 0.001), age (*p* < 0.001), medical specialty (*p* < 0.001), information on monkeypox during undergraduate medical education (*p* < 0.001), and history of COVID-19 (*p* < 0.001), was associated with a good knowledge score about monkeypox (Table 4). Good knowledge scores of monkeypox were more common among female physicians (42.2% vs. 18.8%) than male physicians. Participants aged over 50 and aged 30–49 exhibited a greater degree of good knowledge scores (50% and 48.3%, respectively) compared to those aged 18–29 (18.1%). In terms of medical specialty, physicians in the internal medical sciences division displayed a significantly higher level of knowledge than others (40.7% vs. 19.8). Furthermore, the prevalence of good knowledge was understandably prominent among physicians who had information about human monkeypox during medical education (53.3% vs. 26.9%). Overall, the most frequently reported source of information was social media (51.9%), followed by research articles (48.8%), TV and radio (27.9%), family or friends (15.9%), and books (15.5%). We observed a significant relationship between the category of knowledge and sources of information on monkeypox (*p* < 0.01) (Table 2). The majority of participants with good knowledge acquired information from research articles (76.1%) as their sources of information, compared to those who used social media (37%), books (26.1%), and TV and radio (17.4%).

Most participants (66.7%) over the age of 50 expressed a positive attitude toward monkeypox. This was significantly higher (*p* = 0.005) compared to those of the other age groups of 18–29 years and 30–49 years, as only 33.6% and 48.3% of their participants reached good attitude scores, respectively. A comparatively greater extent of positive attitude was also measured in physicians who learned about monkeypox throughout their medical education (56.7% vs. 37.7%) and who had a history of COVID-19 (45.2% vs. 34.7%). Furthermore, physicians who achieved good knowledge scores exhibited significantly higher positive attitudes than their counterparts (58.7% vs. 33.5%, *p* < 0.001).

### 3.3. Worries from Monkeypox Disease and Vaccination Readiness

Physicians’ monkeypox disease worries, concerns, and willingness to receive vaccines are displayed in Table 3. Currently, 62.5% of participants are more worried about COVID-19 than monkeypox, 20.1% are more concerned about monkeypox, while 17.3% of HCWs are unsure or equally worried about both diseases. Considering participants’ leading causes of worry, 33.6% of respondents are worried about themselves and their families acquiring the infection. This was followed by concern about the progression of the disease into a global pandemic (25.4%) and international flight restrictions (3.2%). We further evaluated participants’ overall generalized anxiety with the GAD7 scale, and their overall mean score was 5.44/21 SD 5.01 points, highlighting mild anxiety levels. While the GAD 7 score was not associated with the knowledge and attitude score, a significantly higher willingness rate was observed during χ2-analysis for the participants with higher GAD-7 at the ≥10 cutoff point (*p* = 0.01)

As shown in Table 4, various factors significantly affect willingness to be vaccinated. These include gender (*p* = 0.027), information on monkeypox during medical education (*p* = 0.001), more worried about monkeypox compared to COVID-19, and history of COVID-19 infection (*p* = 0.021). Nearly one third (31.4%) of the physicians planned to be vaccinated, and 85 (30.0%) refused to be vaccinated against monkeypox infection. When the government offered the vaccination for free, 145 (51.2%) of the participants were willing to be vaccinated. Additionally, a significantly higher willingness rate was observed during chi-square analysis for the participants with better knowledge levels than the poor knowledge-categorized people (47.8% vs. 23.6%, *p* < 0.001), while the positive attitude was not significantly associated with the willingness (34.7% vs. 48.8%, *p* = 0.001)

### 3.4. Logistic Regression Analysis of Knowledge and Willingness to Vaccination

The female participants were almost two times more likely than the male participants to be knowledgeable about monkeypox (95% CI = 1.06, 3.97; *p* = 0.031). Similarly, the knowledgeability of physicians about monkeypox aged 30 and over was found to be significantly higher than that of the participants under the age of 30 (AOR = 2.38, 95% CI = 1.27, 4.44; *p* = 0.007). Furthermore, being physicians in divisions of internal medicine (AOR: 2.07; 95% CI: 1.06–4.03, *p* = 0.033) was significantly associated with a good knowledge score compared to other physicians. A good knowledge score for monkeypox was significantly associated with having information on monkeypox during medical education (AOR: 2.77; 95% CI = 1.36.-5.66; *p* = 0.005) and previous exposure to COVID-19 disease (AOR: 2.72; 95% CI:1.35–5.47; *p* = 0.005). Moreover, physicians who used original scientific articles rather than news on social media as a source of monkeypox virus related information were 3.57 times more likely to be knowledgeable about monkeypox (Table 5).

In multivariate analysis, willingness to be vaccinated for monkeypox was significantly associated with a good knowledge score (AOR: 2.22; 95% CI:1.13–4.33; *p* = 0.019). In addition, those participants who were more concerned about monkeypox compared to COVID-19 (AOR = 4.01, 95% CI = 2.31–8.89; *p* < 0.001) and had information on monkeypox during medical education (AOR = 2.16, 95% CI = 1.10– 4.21; *p* = 0.024) were more likely to get the smallpox vaccine to prevent monkeypox infection once made available.

## 4. Discussion

To the best of our knowledge, this is the first study that assessed the knowledge, attitude, and concerns of Turkish physicians towards human monkeypox and related vaccination and the factors that affected their knowledge level and willingness for vaccination. In our study, while nearly one third of the physicians had good knowledge about monkeypox (32.5%), the proportion of physicians who wanted to get the smallpox vaccine to prevent monkeypox when available was similar (31.4%). Based on our findings, the physicians’ knowledge level about monkeypox was significantly better in women over 30 years of age, working in divisions of internal medicine, having knowledge of monkeypox during medical education, using original articles as sources of information, and having a personal history of COVID-19 infection. We also found physicians with a good knowledge score, more worried about Monkeypox than COVID-19, and having information on monkeypox during medical education were more likely to get the smallpox vaccine to prevent monkeypox viral infection.

The world has faced the tremendous burden of the COVID-19 pandemic for the past two years. While it was not the most severe pandemic in modern history, it has caused unprecedented economic and health disruptions for humans [23]. Hence, the announcement of a recent resurgence of the monkeypox virus has raised considerable concerns about the possibility of developing another pandemic in the shadow of COVID-19. The appropriate and timely response of HCWs is an important prerequisite for tackling the ongoing monkeypox outbreak. The baseline level of monkeypox knowledge must be evaluated in order to achieve this goal, especially among physicians, given their crucial position in mitigating the monkeypox outbreak. In addition, amid the ongoing monkeypox epidemic, physicians represent a key group that may be at risk of acquiring and further transmitting the virus. The evaluation of their knowledge about monkeypox and their confidence levels in early detection, prevention, and management of the potential cases is of utmost value. Subsequently, knowledge gaps can be remedied with appropriate training and education.

In our study, the overall knowledge level among Turkish physicians regarding monkeypox was insufficient for most of the questions. Among the questions pointing to a satisfactory level of knowledge in terms of the awareness were that a virus causes monkeypox, does not only affect males, and shares common features with smallpox. Before the current monkeypox outbreak, similar levels of knowledge on these aspects were previously found in a study conducted among general practitioners in Indonesia [17]. On the contrary, obvious defects in knowledge emerged with different parts of the disease, including transmission, epidemiology, prevention, and treatment. For instance, only a third of the participants correctly knew that a smallpox vaccine was available for monkeypox. Our findings were consistent with studies from Indonesia, Jordan, and Italy, despite the lack of literature that examined HCWs’ knowledge of monkeypox [17,19,24]. In the 2019 study by Harapan et al., 158 out of 432 (36.5%) general practitioners reported having good knowledge [17]. Italian doctors also demonstrated similarly low levels of knowledge, according to a recent study by Matteo Riccò et al. [19]. In another study, Jordanian HCWs had <50% correct answers on 4 of 11 monkeypox knowledge items versus >70% correct answers on only 3 items [24]. Low levels of monkeypox knowledge were also reported among the general population in Lebanon and Saudi Arabia [25,26].

There are conflicting findings in studies that examine how gender affects the level of knowledge of monkeypox. Our study found that males possessed low levels of general knowledge about the monkeypox virus. Consistent with our research, Alshahrani et al. achieved comparable results, reporting that female Saudi physicians had significantly better knowledge scores about the monkeypox virus than their male counterparts [18]. However, our study’s findings contradicted those of Sallam et al. They observed that male HCWs were significantly more knowledgeable about monkeypox than their female counterparts [24]. Good knowledge about monkeypox increased with age in our study. Possible explanations for the comparatively low degree of monkeypox knowledge observed in this study include the young mean age of the study sample (33 years). This represents the majority of participants living in the post-smallpox eradication era, with a declining focus on poxviruses in training and education [27,28]. This is also associated with the higher level of knowledge of those who had heard of monkeypox during their medical education.

The spread of monkeypox continues to add to the current burden of anxiety experienced by the public and HCWs. According to a recent study, over 60% of the general population thought the COVID-19 pandemic was more worrying than monkeypox, while 37.4% reported they were more worried about monkeypox [20]. In our study, only 20.1% of physicians were more concerned with monkeypox than COVID-19. As a result, our participants did not have serious concerns about monkeypox. However, self and family worry about monkeypox infection and the current global outbreak is among the main reasons for concerns about the present monkeypox virus (33.6% and 25.4%, respectively). This worry is presumably linked to their recent experience with the COVID-19 pandemic.

Vaccination against monkeypox at this stage of the disease is a challenging decision for local government officials, international health organizations, and healthcare policymakers. Two vaccines are currently approved that can protect against monkeypox. Although there is no data yet on the efficacy of either vaccine in the current monkeypox outbreak, ACAM2000 is a second-generation smallpox vaccine, and IMVANEX vaccine is a replication-deficient live vaccine produced from a modified vaccinia Ankara virus, which has previously been given to HCWs who were involved in investigating monkeypox outbreaks [29,30]. In light of recent outbreaks and the reemergence of the disease, advocacy for vaccinating HCWs against monkeypox requires further research. The current study did not find any relationship between HCWs’ different demographics and vaccine acceptance. However, those who knew about monkeypox during medical training and those who perceived it more worrisome than COVID-19 were more likely to support vaccination against it. This is consistent with previous research on the subject because perceiving the disease as severe was associated with increased willingness for vaccination [20,31,32,33]. Our cohort has shown that those with good knowledge scores were significantly more supportive of vaccination against monkeypox; the same phenomenon has been reported in public with the COVID-19 vaccine, even after two years of the pandemic [21]. In the study by Meo et al., 71.4% of participants were aware of the role of the smallpox vaccine in protecting against monkeypox [34], and only 58.6% of participants in the Italian study favored the administration of variola vaccines to prevent monkeypox in some way [19]. In contrast to these high rates, in our cohort, only 39.2% were aware of the smallpox vaccine’s role in protecting against monkeypox. Given the importance of the physician’s role in vaccination, these findings highlight the need for physicians to develop their knowledge and attitude to make strong vaccination recommendations to their patients.

The strength of the current study stems from the fact that it was the first to evaluate physicians’ knowledge and attitudes about the monkeypox outbreak and vaccines in a selected group of physicians in a large tertiary care hospital in Turkey. The findings of this study may help develop awareness campaigns to further improve the knowledge of virus emergence and diseases. However, a few limitations affect the accurate interpretation of the study results. Firstly, we could not conduct previously validated knowledge and attitude scales, as there was no valid questionnaire for the Turkish population during the study. Second, because it was completed online and used convenience sampling, this study was susceptible to selection bias.

Another critical point was the cross-sectional study design; the cause-and-effect link cannot be attributed to the regression model’s findings. Another limitation is the scarcity of such cases in Turkey, and the fact that physicians are unfamiliar with the disease, which may influence their knowledge, attitude, and worries. As the study included data from a single center and a small group of 283 physicians, it can hardly be considered fully representative of the national level. Longitudinal studies based on face-to-face interviews with randomized and larger samples are needed and may validate our findings.

## 5. Conclusions

Monkeypox disease knowledge among physicians is critical in minimizing the disease burden and tackling viral infectious diseases at regional and global levels. The present study pointed out that physicians in Turkey have unsatisfactory levels of knowledge about the emerging monkeypox. The majority of physicians had a negative attitude and stated that they were reluctant to vaccinate if there was a vaccination campaign to combat the spread of monkeypox. Therefore, research activities to close the knowledge gap in the field must be stepped up immediately to halt current and future epidemics and optimize our preparedness to confine and combat monkeypox and zoonotic illnesses. This could pave the foundation for policymakers on how to support monkeypox vaccination strategies and how to address potential vaccine hesitancy when needed.

## Figures and Tables

**Table 1:** Responses of physicians who participated in the study to the knowledge questions about the monkeypox disease (n = 283).

Statement	Response	Total N (%)
1. Monkeypox is a new infection that appeared this year 2022 *	Correct	36 (12.7)
	Do not know	41 (14.5)
	Incorrect	206 (72.8)
2. Monkeypox is a sexually transmitted disease *	Correct	89 (31.4)
	Do not know	45 (15.9)
	Incorrect	149 (52.7)
3. Monkeypox is transmitted to humans through the bites and scratches from infected animals.	Correct	119 (42)
	Do not know	83 (29.3)
	Incorrect	81 (28.6)
4. Monkeypox is easily transmitted from one person to another *	Correct	132 (46.6)
	Do not know	47 (16.6)
	Incorrect	104 (36.7)
5. Monkeypox is spread by droplets (coughing and sneezing)	Correct	134 (47.3)
	Do not know	49 (17.3)
	Incorrect	100 (35.3)
6. Monkeypox only affects males *	Correct	1 (0.4)
	Do not know	12 (4.2)
	Incorrect	270 (95.4)
7. Hand sanitizers and face masks are important in preventing monkeypox	Correct	196 (69.3)
	Do not know	54 (19.1)
	Incorrect	33 (11.7)
8. Monkeypox is prevalent in the Middle Eastern countries *	Correct	41 (14.5)
	Do not know	98 (34.6)
	Incorrect	144 (50.9)
9. Monkeypox is a bacterial disease infection *	Correct	1 (0.4)
	Do not know	9 (3.2)
	Incorrect	273 (96.5)
10. Monkeypox and smallpox have similar signs and symptoms	Correct	212 (74.9)
	Do not know	54 (19.1)
	Incorrect	17 (6.0)
11. Monkeypox and chickenpox have similar signs and symptoms	Correct	160 (56.5)
	Do not know	67 (23.7)
	Incorrect	56 (19.8)
12. Papules and vesicles on the skin are one of the signs or symptoms of human monkeypox	Correct	246 (86.9)
	Do not know	30 (10.6)
	Incorrect	7 (2.5)
13. Lymphadenopathy (swollen lymph nodes) is one clinical sign or symptom that could be used to differentiate monkeypox and smallpox cases	Correct	116 (41)
	Do not know	135 (47.7)
	Incorrect	32 (11.3)
14. There is a specific vaccine for monkeypox *	Correct	33 (11.7)
	Do not know	85 (30.0)
	Incorrect	165 (58.3)
15. Monkeypox virus causes milder disease in children (<14 years) than adults *	Correct	72 (25.4)
	Do not know	163 (57.6)
	Incorrect	48 (17.0)
16. What is the monkeypox virus case fatality rate in the general population?	0–11%	145 (51.2)
	12–20%	130 (45.9)
	21–30%	5 (1.8)
	31–40%	2 (0.7)
	Do not know	1 (0.4)
17. There is a specific treatment for monkeypox *	Correct	12 (4.2)
	Do not know	99 (35.0)
	Incorrect	172 (60.8)
18. The chickenpox vaccine I got in childhood protects me from monkeypox *	Correct	46 (16.3)
	Do not know	79 (27.9)
	Incorrect	158 (55.8)
19. There is a smallpox vaccine that can be used for monkeypox	Correct	112 (39.6)
	Do not know	118 (41.7)
	Incorrect	53 (18.7)
20. People can be vaccinated after exposure to monkeypox virus to help prevent monkeypox disease	Correct	58 (20.5)
	Do not know	142 (50.2)
	Incorrect	83 (29.3)

* Human monkeypox knowledge items that are marked with an asterisk represent incorrect statements.

**Table 2:** Association between baseline characteristics and knowledge score about monkeypox disease among physicians (n = 283).

Variables		n (%)	Good Knowledge; n (%)	Poor Knowledge; n (%)	*p* Value
Age					
	18–29 years	149 (52.7)	27 (29.3)	122 (63.9)	<0.001
	30–49 years	116 (41)	56 (60.9)	60 (31.4)	
	50 years and above	18 (6.4)	9 (9.8)	9 (4.7)	
Gender					
	Female	166 (58.7)	70 (76.1)	96 (50.3)	<0.001
	Male	117 (41.3)	22 (23.9)	95 (49.7)	
Medical specialty					
	Intern Doctor	35 (12.4)	5 (5.4)	30 (15.7)	0.004
	General Practitioner	29 (10.2)	8 (8.7)	21 (11)	
	Basic Sciences Division	10 (3.5)	3 (3.3)	7 (3.7)	
	Internal Medical Sciences Division	172 (60.8)	70 (76.1)	102 (53.4)	
	Surgical Sciences Division	37 (13.1)	6 (6.5)	32 (16.2)	
Medical practice experience					
	Less than 1 year	58 (20.5)	9 (9.8)	49 (25.7)	<0.001
	1–5 years	110 (38.9)	27 (29.3)	83 (43.5)	
	5–10 years	35 (12.4)	14 (15.2)	21 (11.0)	
	10–20 years	55 (19.4)	30 (32.6)	25 (13.1)	
	More than 20 years	25 (8.8)	12 (13.0)	13 (6.8)	
Information on monkeypox during medical education					
	No	223 (78.8)	60 (65.2)	163 (85.3)	<0.001
	Yes	60 (21.2)	32 (34.8)	28 (14.7)	
First time you heard information about monkeypox					
	Within several days or weeks ago	13 (4.6)	2 (2.2)	11 (5.8)	0.187
	Within the last month or later	267 (94.3)	90 (97.8)	177 (92.7)	
	I did not hear about it	3 (1.1)	0 (0.0)	3 (1.6)	
Personal history of COVID-19					
	No	95 (33.6)	17 (18.5)	78 (40.8)	<0.001
	Yes	188 (66.4)	75 (81.5)	113 (59.2)	
COVID-19 vaccination history					
	0 to 3 COVID-19 vaccine shots	80 (28.3)	22 (23.9)	58 (30.4)	0.259
	4 or more COVID-19 vaccine shots	203 (71.7)	70 (76.1)	133 (69.6)	
Sources of monkeypox virus related information (multiple choices)					
	TV and radio	79 (27.9)	16 (17.4)	63 (33.0)	0.007
	Social media	147 (51.9)	34 (37)	113 (59.2)	0.001
	Family or friend	45 (15.9)	9 (9.8)	36 (18.8)	0.057
	Books	44 (15.5)	24 (26.1)	20 (10.5)	0.001
	Research articles	138 (48.8)	70 (76.1)	68 (35.6)	<0.001

**Table 3:** Respondents’ attitudes, perceptions, and beliefs about monkeypox disease (N = 283).

Variables		n (%)
Worry from Monkeypox compared to COVID-19		
	More worried with Monkeypox	57 (20.1)
	More worried with the COVID-19	177 (62.5)
	Similar worries with Monkeypox and COVID-19	49 (17.3)
Main reasons for your worry from monkeypox disease		
	Self and family worry of Monkeypox infection	95 (33.6)
	Worry from another worldwide 3pandemic	72 (25.4)
	Worried that Monkeypox might surge to cause a national lockdown	8 (2.8)
	Concerned that an international flight suspension happens	9 (3.2)
	Other worries	5 (1.8)
	Not worried	94 (33.2)
GAD7 score, mean (SD)		5.44 (5.01)
	Very low Anxiety	142 (50.2)
	Mild Anxiety	93 (32.9)
	Moderate Anxiety	26 (9.2)
	High Anxiety	22 (7.8)
Overall Monkeypox Disease Knowledge score, Mean (SD) maximum score = 20		11.26 (3.67)
	Poor Knowledge	191 (67.5)
	Good Knowledge	92 (32.5)
Attitude, Mean (SD) maximum score = 10		6.10 (2.05)
	Negative	165 (58.3)
	Positive	118 (41.7)
Intentions toward monkeypox vaccination uptake		
	Willing	89 (31.4)
	Hesitated	109 (38.5)
	Not willing	85 (30.0)
Worry about the possible side effects of vaccination against monkeypox infection		
	Agree	48 (17.0)
	Neither agree or disagree	91 (32.2)
	Disagree	144 (50.9)
If the country provides the vaccination against monkeypox infection for free, I am willing to be vaccinated.		
	Agree	145 (51.2)
	Neither agree or disagree	109 (38.5)
	Disagree	29 (10.2)

Abbreviations: GAD-7, General Anxiety Disorder-7; SD, standard deviation.

**Table 4:** Chi-square (x2) test of baseline characteristics of physicians who have good knowledge, positive attitude, and willingness of getting monkeypox vaccine.

Variables		Good Knowledge			Positive Attitude			Having Willingness		
		N	%	*p*-Value	N	%	*p*-Value	N	%	*p*-Value
Overall	Categories	92	32.5	-	118	41.7	-	89	31.4	-
Gender	Female	70	42.2	<0.001	69	41.6	0.958	61	36.7	0.027
	Male	22	18.8		49	41.9		28	23.9	
Age	18–29 years	27	18.1	< 0.001	50	33.6	0.005	40	26.8	0.089
	30–49 years	56	48.3		56	48.3		40	34.5	
	50 years and above	9	50		12	66.7		9	50.0	
Medical specialty	Intern Doctor	5	14.3	0.004	12	34.3	0.097	13	37.1	0.251
	General Practitioner	8	27.6		8	27.6		13	44.8	
	Basic Sciences Division	3	30.0		2	20		1	10.0	
	Internal Medical Sciences Division	70	40.7		82	47.7		51	29.7	
	Surgical Sciences Division	6	16.2		14	37.8		11	29.7	
Medical practice experience	Less than 1 year	9	15.5	<0.001	17	29.3	0.053	20	34.5	0.201
	1–5 years	27	24.5		43	39.1		30	27.3	
	5–10 years	14	40		15	42.9		7	20.0	
	10–20 years	30	54.5		28	50.9		22	40.0	
	More than 20 years	12	48		15	60.0		10	40.0	
Information on monkeypox during medical education	Yes	32	53.3	<0.001	34	56.7	0.012	30	50	0.001
	No	60	26.9		84	37.7		59	26.5	
Previous exposure to COVID- 19 disease	Yes	75	39.9	<0.001	85	45.2	0.098	68	36.2	0.021
	No	17	17.9		33	34.7		21	22.1	
More worried from monkeypox compared to COVID19	Yes	23	40.4	0.159	31	54.4	0.035	34	59.6	<0.001
	No	69	30.5		87	38.5		55	24.3	
Sources of monkeypox virus related information (multiple choices)	TV and radio	16	20.3	0.006	29	36.7	0.347	25	31.6	0.965
	Social media	34	23.1	<0.001	56	38.1	0.228	45	30.6	0.753
	Family or friend	9	20.0	0.051	18	40.0	0.870	13	28.9	0.730
	Books	24	54.5	0.001	18	40.9	0.908	24	54.5	<0.001
	Research articles	70	50.7	<0.001	68	49.3	0.016	55	39.9	0.003
GAD-7 Score	Very low Anxiety	43	30.3	0.719	59	41.5	0.923	41	28.9	0.058
	Mild Anxiety	34	36.6		37	39.8		25	26.9	
	Moderate Anxiety	9	34.6		12	46.2		13	50	
	High Anxiety	6	27.3		10	45.5		10	45.5	
Knowledge	Good	-	-	-	54	58.7	<0.001	44	47.8	<0.001
	Poor	-	-	-	64	33.5		45	23.6	
Attitude	Positive	-	-	-	-	-	-	41	34.7	0.364
	Negative	-	-	-	-	-	-	48	29.1	

Abbreviations: GAD-7, General Anxiety Disorder-7.

**Table 5:** Logistic regression analysis of knowledge and willingness to get monkeypox vaccine of physicians of Turkey.

Variables	Knowledge				Willingness			
	AOR	*p*-Value	95% CI		AOR	*p*-Value	95% CI	
Gender (female vs. male)	2.05	0.031	1.06	3.97	1.38	0.308	0.74	2.58
Age (≥30 vs. <30 years)	2.38	0.007	1.27	4.44	1.04	0.885	0.85	3.82
Medical specialty (internal medical science divisions vs. others)	2.07	0.033	1.06	4.03	0.66	0.188	0.35	1.22
Information on monkeypox during medical education (yes vs. no)	2.77	0.005	1.36	5.66	2.16	0.024	1.10	4.21
Previous exposure to COVID- 19 disease (yes vs. no)	2.72	0.005	1.35	5.47	1.68	0.113	0.88	3.23
More worried from monkeypox compared to COVID19 (yes vs. no)	1.16	0.687	0.54	2.49	4.53	<0.001	2.31	8.89
GAD-7 Score (≥10 vs. <10 )	1.10	0.818	0.47	2.58	1.80	0.121	0.85	3.82
Use of original article as a source of monkeypox virus related information (yes vs. no)	3.57	<0.001	1.89	6.73	1.57	0.152	0.84	2.94
Attitude (positive vs. negative),	1.97	0.03	1.06	3.67	1.25	0.458	0.68	2.29
Knowledge score (good vs. poor)	-	-	-	-	2.22	0.019	1.13	4.33

Abbreviations: AOR, adjusted odds ratio; CI, confidence interval; GAD-7, General Anxiety Disorder-7.

## Data Availability

The data that support the findings of this study are available on request from the corresponding author. The data is not publicly available due to privacy or ethical restrictions.

## Supplementary Materials

The following supporting information can be downloaded at: https://www.mdpi.com/article/10.3390/vaccines11010019/s1, Table S1: English Translation of the Questionnaire; Table S2: Respondents’ attitudes, beliefs and concerns about monkeypox disease (n = 283).
