# Supplementary material for: Knowledge and Attitudes of Turkish Physicians towards Human Monkeypox Disease and Related Vaccination: A Cross-Sectional Study
Source: Vaccines (Basel). 2022 Dec 21;11(1):19. doi: 10.3390/vaccines11010019 (PMC9863926; doi:10.3390/vaccines11010019)
Supplement: Supplementary file 1 [file vaccines-11-00019-s001.zip › vaccines-2057043-supplementary.pdf]

Supplementary Table S1. English Translation of the Questionnaire.

If you have any concerns, questions, and complaints about this study, its risks, benefits, and procedures contact the Protocol Director, Dr. Meliha Cagla Sonmezer at (+90) 505 892 32 78. *Independent Contact:* If you are not satisfied with how this study is being conducted, or if you have any concerns, complaints, or general questions about the research or your rights as a participant, please contact the Hacettepe University Institutional Review Board to speak to someone independent of the research team at (+90) 312-305-1082 or email at goetik@hacettepe.edu.tr. If you agree to participate in this research, please select "Yes" below.	
**Informed consent**	
We have conducted a survey entitled "**Knowledge and Attitudes of Turkish Physicians towards Human Monkeypox Disease and Related Vaccination: A Cross-Sectional Study**". The results of the study will represent the knowledge and attitudes of Physicians towards Monkeypox infection vaccinations. The survey may take 10-15 minutes. Nowhere in the survey, you won't be asked for your personal information. All of your information will be kept secret. You have the right to participate or deny, and during the time of participation, you can withdraw yourself from responding. The study will not be benefitted you by money or other compensations but the outcome of the study may consider by the policymakers and take initiative for monkeypox vaccinations in Turkey. Thank you for your participation.	
Are you willing to participate in the survey?	o Yes<br>o No
**Section 1: Sociodemographic data**	
1. Birth year	………………………..
2. If you were born before 1980, have you been vaccinated against smallpox or do you have a scar on the left upper arm?	o yes i am vaccinated<br>o no i'm not vaccinated<br>o I don't know my vaccination status<br>o I don't have a vaccination scar<br>o I have a vaccination scar
3. Gender	o Female<br>o Male
4. Medical Specialty	o General practitioner<br>o Specialist<br>o Internal medicine<br>o Pulmonology<br>o Infectious Disease<br>o Family medicine<br>o Pediatrics<br>o Emergency medicine<br>o Intensive care<br>o Surgery<br>o Other (write in text)
5. Medical practice experience	o Less than 1 year,<br>o 1-5 years<br>o 5-10 years<br>o 10-20 years<br>o >20 years
6. Received information of human Monkeypox during medical education	o Yes

	○ No
7. Heard about human Monkeypox before	○ Yes<br>○ No
8. First time you heard information about Monkeypox	○ Within severaldays or weeks ago<br>○ Within last month or later<br>○ I did not hear about it
9. Have you been affected by the COVID-19 disease?	○ Yes<br>○ No
10.Worry from Monkeypox compared to COVID-19	○ Much worried with the COVID-19<br>○ Much worried with Monkeypox<br>○ Similar worries with Monkeypox and COVID-19
11. If you much worried with Monkeypox;<br>Main reasons for your worry from Monkeypox disease	○ Self and family worry of Monkeypox infection<br>○ Worry from another worldwide pandemic<br>○ Worried that Monkeypox might surge to cause a national lockdown<br>○ Concerned that an international flight suspension happens<br>○ Other worries
12.Have you gotten all COVID-19 vaccine shots ?	○ Yes<br>○ No
13. Which of the following sources will you choose to derive monkeypox virus -related information? (multiple choices)	○ TV and radio<br>○ Social media<br>○ Family or friend<br>○ Books<br>○ Research articles
**Section 2: Knowledge**	
1. Monkeypox is a new infection that appeared this year 2022	○ Correct<br>○ Incorrect<br>○ Do not know
2. Monkeypox is a sexually transmitted disease	○ Correct<br>○ Incorrect<br>○ Do not know
3. Monkeypox is transmitted to humans through the bites and scratches from infected animals.	○ Correct<br>○ Incorrect<br>○ Do not know

4. Monkeypox is easily transmitted from one person to another	o Correct<br>o Incorrect<br>⊖ Do not know
5.Monkeypox is spread by droplets (coughing and sneezing)	o Correct<br>o Incorrect<br>⊖ Do not know
6. Monkeypox only affects males.	o Correct<br>o Incorrect<br>⊖ Do not know
7. Hand sanitizers and face masks are important in preventing monkeypox	o Correct<br>o Incorrect<br>⊖ Do not know
8. Monkeypox is prevalent in middle eastern countries	o Correct<br>o Incorrect<br>⊖ Do not know
9. Monkeypox is a bacterial disease infection	o Correct<br>o Incorrect<br>⊖ Do not know
10. Monkeypox and smallpox have similar signs and symptoms	o Correct<br>o Incorrect<br>⊖ Do not know
11. Monkeypox and chickenpox have similar signs and symptoms	o Correct<br>o Incorrect<br>⊖ Do not know
12. Papules, rashes and vesicles on the skin are one of the signs or symptoms of human monkeypox	o Correct<br>o Incorrect<br>⊖ Do not know
13. Lymphadenopathy (swollen lymph nodes) is one clinical sign or symptom that could be used to differentiate monkeypox and smallpox cases	o Correct<br>o Incorrect<br>⊖ Do not know
14. There is a specific vaccine for monkeypox	o Correct<br>o Incorrect<br>⊖ Do not know
15. Monkeypox virus causes milder disease in children (<14 years) than adults	o Correct<br>o Incorrect<br>o Do not know

16. What is the monkeypox virus case fatality rate in the general population?	o 0-11 %<br>o 12-20 %<br>o 21-30 %<br>o 31-40 %<br>o Do not know
17. There is a specific treatment for monkeypox	o Correct<br>o Incorrect<br>o Do not know
18. The chickenpox vaccine I got in childhood protects me from monkeypox	o Correct<br>o Incorrect<br>o Do not know
19. There is a smallpox vaccine that can be used for monkeypox	o Correct<br>o Incorrect<br>o Do not know
20. People can be vaccinated after exposure to monkeypox virus to help prevent monkeypox disease	o Correct<br>o Incorrect<br>o Do not know
**Section 3: Attitude**	
1. I am confident that the world's population can control the monkeypox worldwide	o Disagree<br>o Undecided<br>o Agree
2.I am interested in learning more about monkeypox	o Disagree<br>o Undecided<br>o Agree
3. I think that it is not dangerous to travel to the countries epidemic with monkeypox	o Disagree<br>o Undecided<br>o Agree
4. I think monkeypox will not affect social and economic life like the COVID- 19 pandemic	o Disagree<br>o Undecided<br>o Agree
5. I think that monkeypox cannot add new burden on healthcare system of the affected countries	o Disagree<br>o Undecided<br>o Agree
**4. Vaccination Willingness**	
1. I'm considering getting the smallpox vaccine to prevent monkeypox viral infection	o Disagree<br>o Undecided<br>o Agree

2. The monkeypox infection has been alleviated, and there is no need to be vaccinated against monkeypox infection.	o Disagree<br>o Undecided<br>o Agree
3. I am worried about the possible side effects of vaccination against monkeypox infection	o Disagree<br>o Undecided<br>Agree
4. The recommendation for the vaccination against monkeypox infection by doctors, the community and other professionals has a great influence on me.	o Disagree<br>o Undecided<br>o Agree
5. If the country provides the vaccination against monkeypox infection for free, I am willing to be vaccinated.	o Disagree<br>o Undecided<br>o Agree
**Section 5. Anxiety**	
**Over the last 2 weeks, how often have you been bothered by the following problems?**	
1. Feeling nervous, anxious or on edge	o Not at all<br>o Several days<br>o More than half the days<br>o Nearly every day
2. Not being able to stop or control worrying	o Not at all<br>o Several days<br>o More than half the days<br>o Nearly every day
3. Worrying too much about different things	o Not at all<br>o Several days<br>o More than half the days<br>o Nearly every day
4. Trouble relaxing	o Not at all<br>o Several days<br>o More than half the days<br>o Nearly every day
5. Being so restless that it is hard to sit still	o Not at all<br>o Several days<br>o More than half the days<br>o Nearly every day
6. Becoming easily annoyed or irritable	o Not at all<br>o Several days<br>o More than half the days<br>o Nearly every day
7. Feeling afraid as if something awful might happen	o Not at all

	o Several days<br>o More than half the days<br>o Nearly every day

Supplementary Table S2. Respondents' attitudes, beliefs and concerns about Monkeypox disease (n = 283).

Statement	Response	Total N (%)
A1. I am confident that the world's population can control the monkeypox worldwide	Agree	126 (44.5)
	Neither agree or disagree	124 (43.8)
	Disagree	33 (11.7)
A2. I am interested in learning more about monkeypox	Agree	229 (80.9)
	Neither agree or disagree	37(13.1)
	Disagree	17 (6.0)
A3. I think that it is not dangerous to travel to the countries epidemic with monkeypox	Agree	77 (27.2)
	Neither agree or disagree	85 (30.0)
	Disagree	121 (42.8)
A4. I think monkeypox will not affect social and economic life like the COVID- 19 pandemic	Agree	163 (57.6)
	Neither agree or disagree	73 (25.8)
	Disagree	47 (16.6)
A5. I think that monkeypox cannot add new burden on healthcare system of the affected countries	Agree	58 (20.5)
	Neither agree or disagree	72 (25.4)
	Disagree	153 (54.1)
**The** ***General Anxiety Disorder 7*****-item** ***scale***		
1. Feeling nervous, anxious or on edge	Not at all	93 (32.9)
	Several days	126 (44.5)
	More than half the days	28 (9.9)
	Nearly every day	36 (12.7)
2. Not being able to stop or control worrying	Not at all	149 (52.7)
	Several days	99 (35)
	More than half the days	23 (8.1)
	Nearly every day	12 (4.2)
3. Not being able to stop or control worrying	Not at all	87 (30.7)
	Several days	145 (51.2)
	More than half the days	27 (9.5)
	Nearly every day	24 (8.5)
4. Trouble relaxing	Not at all	117 (41.3)
	Several days	120 (42.4)
	More than half the days	24 (8.5)
	Nearly every day	22 (7.8)
5. Being so restless that it is hard to sit still	Not at all	167 (59.0)
	Several days	84 (29.7)
	More than half the days	19 (6.7)
	Nearly every day	13 (4.6)
6. Becoming easily annoyed or irritable	Not at all	124 (43.8)
	Several days	115 (40.6)
	More than half the days	23 (8.1)
	Nearly every day	21 (7.4)
7. Feeling afraid as if something awful might happen	Not at all	155 (54.8)
	Several days	94 (33.2)
	More than half the days	17 (6.0)
	Nearly every day	17 (6.0)

A1. I am confident that the world's population can control the monkeypox worldwide	Agree	126 (44.5)
	Neither agree or disagree	124 (43.8)
	Disagree	33 (11.7)
A2. I am interested in learning more about monkeypox	Agree	229 (80.9)
	Neither agree or disagree	37(13.1)
	Disagree	17 (6.0)
A3. I think that it is not dangerous to travel to the countries epidemic with monkeypox	Agree	77 (27.2)
	Neither agree or disagree	85 (30.0)
	Disagree	121 (42.8)
A4. I think monkeypox will not affect social and economic life like the COVID- 19 pandemic	Agree	163 (57.6)
	Neither agree or disagree	73 (25.8)
	Disagree	47 (16.6)
A5. I think that monkeypox cannot add new burden on healthcare system of the affected countries	Agree	58 (20.5)
	Neither agree or disagree	72 (25.4)
	Disagree	153 (54.1)
**The *General Anxiety Disorder* 7-item *scale***		
1. Feeling nervous, anxious or on edge	Not at all	93 (32.9)
	Several days	126 (44.5)
	More than half the days	28 (9.9)
	Nearly every day	36 (12.7)
2. Not being able to stop or control worrying	Not at all	149 (52.7)
	Several days	99 (35)
	More than half the days	23 (8.1)
	Nearly every day	12 (4.2)
3. Not being able to stop or control worrying	Not at all	87 (30.7)
	Several days	145 (51.2)
	More than half the days	27 (9.5)
	Nearly every day	24 (8.5)
4. Trouble relaxing	Not at all	117 (41.3)
	Several days	120 (42.4)
	More than half the days	24 (8.5)
	Nearly every day	22 (7.8)
5. Being so restless that it is hard to sit still	Not at all	167 (59.0)
	Several days	84 (29.7)
	More than half the days	19 (6.7)
	Nearly every day	13 (4.6)
6. Becoming easily annoyed or irritable	Not at all	124 (43.8)
	Several days	115 (40.6)
	More than half the days	23 (8.1)
	Nearly every day	21 (7.4)
7. Feeling afraid as if something awful might happen	Not at all	155 (54.8)
	Several days	94 (33.2)
	More than half the days	17 (6.0)
	Nearly every day	17 (6.0)
